# Supplementary material for: Visualization Analysis of Small Extracellular Vesicles in the Application of Bone-Related Diseases
Source: Cells. 2024 May 24;13(11):904. doi: 10.3390/cells13110904 (PMC11171653; doi:10.3390/cells13110904)
Supplement: Supplementary file 1 [file cells-13-00904-s001.zip › cells-2983502-supplementary.pdf]

Supporting Information for

Visualization analysis of small extracellular vesicles in the application of bone-related diseases

Xinjiani Chen[a,b,1], Ning Yang[a,1], Bailei Li[a,b], Xinyu Gao[a], Yayu Wang[a,b], Qin Wang[a,b], Xiaojun Liu[a,c,d], Zhen Zhang[a,c,*], Rongqing Zhang[a,b,c*]

[a]Department of Biotechnology and Biomedicine, Yangtze Delta Region Institute of Tsinghua University, Jiaxing, Zhejiang Province 314006, China
[b]Ministry of Education Key Laboratory of Protein Sciences, School of Life Sciences, Tsinghua University, Beijing 100084, China
[c]Zhejiang Provincial Key Laboratory of Applied Enzymology, Yangtze Delta Region Institute of Tsinghua University, 705 Yatai Road, Jiaxing 314006, PR China
[d]Taizhou Innovation Center, Yangtze Delta Region Institute of Tsinghua University, Zhejiang 318000, China
* Correspondence: rqzhang@mail.tsinghua.edu.cn; zhangzhen@tsinghua-zj.edu.cn
[1]These authors contributed equally to this work.

This PDF file includes:

Supplementary Figures S1 to S6
Supplementary Tables 1-3

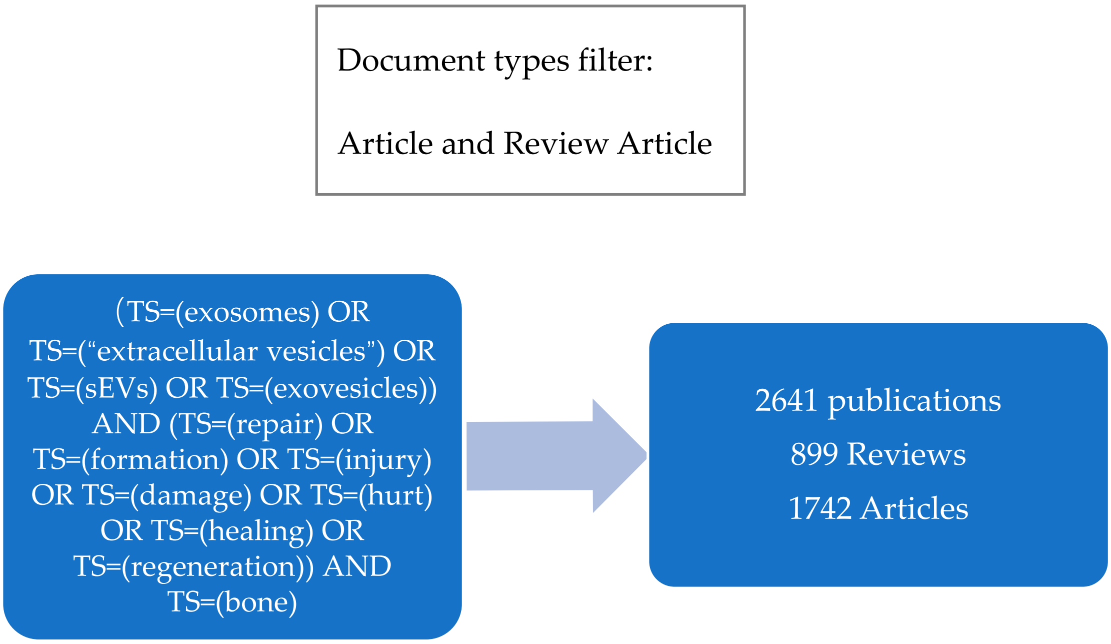

Figure S1 Search terms and results at WOSCC.

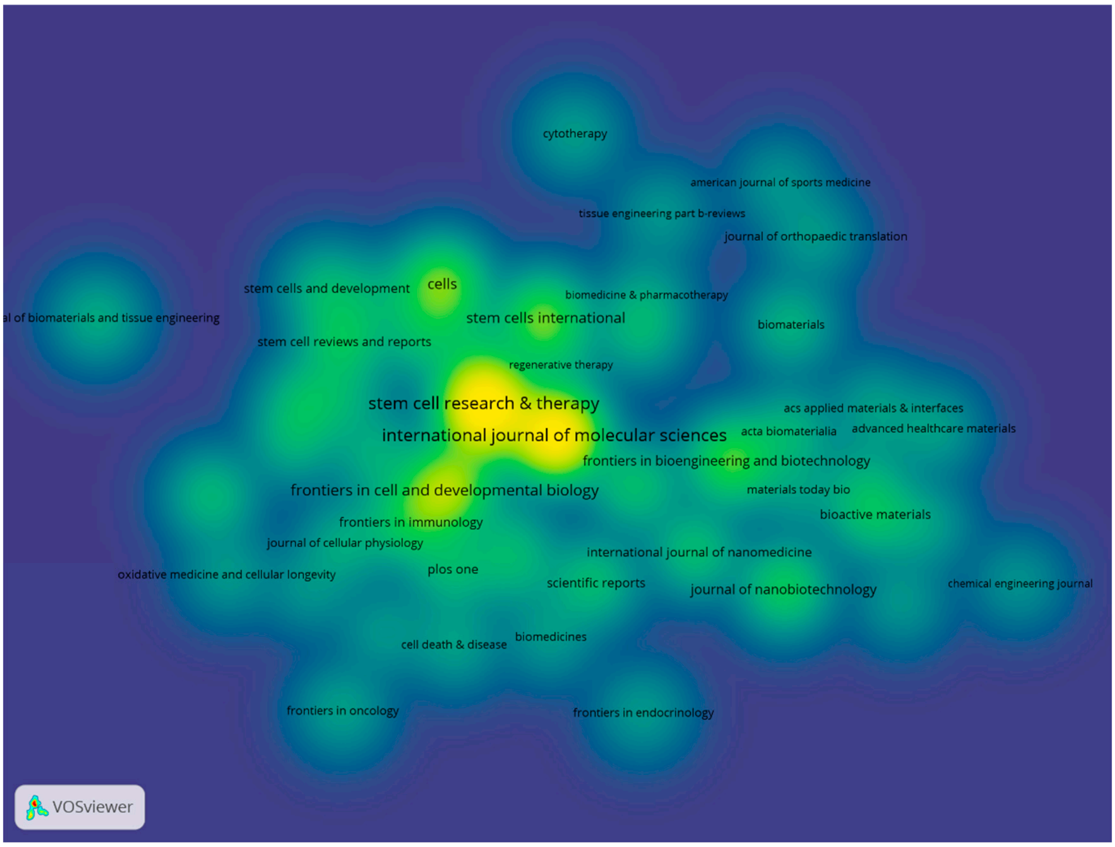

Figure S2 Density visualization of journals conducted by VOSviewer with a threshold of 10.

Table S1

Top 10 journals with the most citations extracted from VOSviewer

Rank	Sources	Document	Citations	Total link strength
1	Stem Cell Research & Therapy	124	8095	1415
2	International Journal of Molecular Science	127	3825	959
3	Stem Cells	18	3380	435
4	Stem Cells Translational Medicine	30	3314	507
5	Stem Cells International	52	2232	540
6	Theranostics	27	1843	291
7	Scientific Reports	25	1813	403
8	Frontiers in Cell and Developmental Biology	68	1690	594
9	Biomatierals	20	1675	383
10	Plos One	22	1630	281

Table S2

Top 10 authors ranked by publication numbers and Top 10 co-cited authors ranked by citations within the publications by CiteSpace

Rank	Publication	Author	Rank	Citations	Centra lity	Co-cited Author
1	16	Camussi, Giovanni	1	519	0.24	THÉRY C
2	13	Tian, Weidong	2	438	0.21	LAI RC
3	12	Liu, Wei	3	366	0.03	ZHANG Y
4	11	Wang, Yan	4	358	0.02	PHINNEY DG

5	11	Ravindran, Sriram	5	337	0.09	DOMINIC I M
6	10	Zhang, Yi	6	323	0.03	VALADI H
7	9	Wang, Xin	7	320	0.08	ZHANG B
8	9	Liu, Guohui	8	309	0.03	RAPOSO G
9	9	Chen, Lang	9	284	0.01	WANG Y
10	8	Liu, Chang	10	278	0.1	ZHANG JY

Table S3

Lokta's law produced by Bibliometrix

Documents written	N. of Authors	Proportion of Authors
1	8946	0.747
2	1669	0.139
3	607	0.051
4	287	0.024
5	139	0.012
6	97	0.008
7	53	0.004
8	40	0.003
9	29	0.002
10	24	0.002

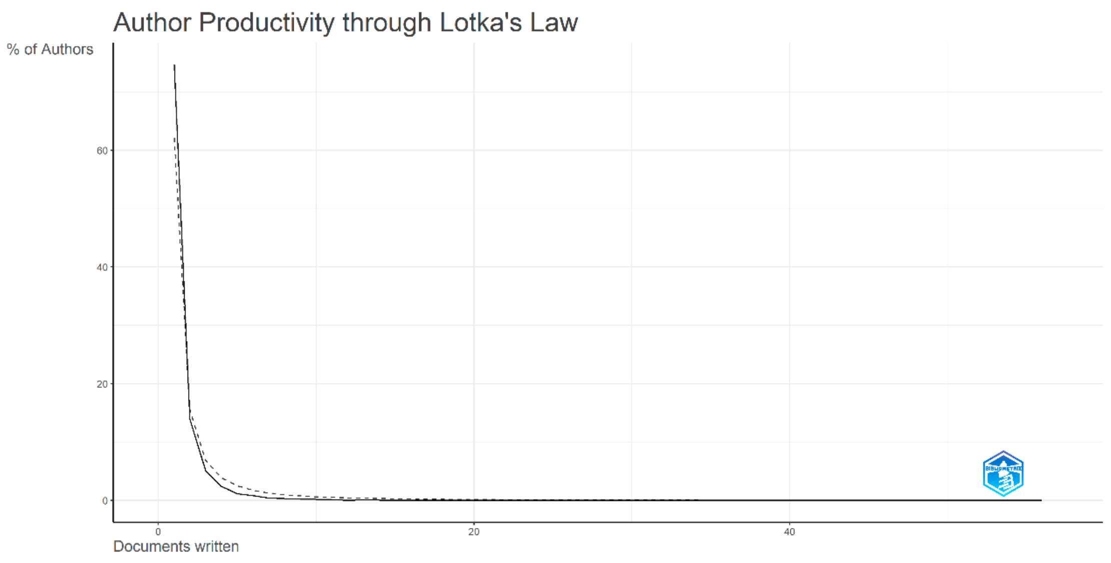

Figure S3 Author productivity through Lotka's law by Bibliometrix.

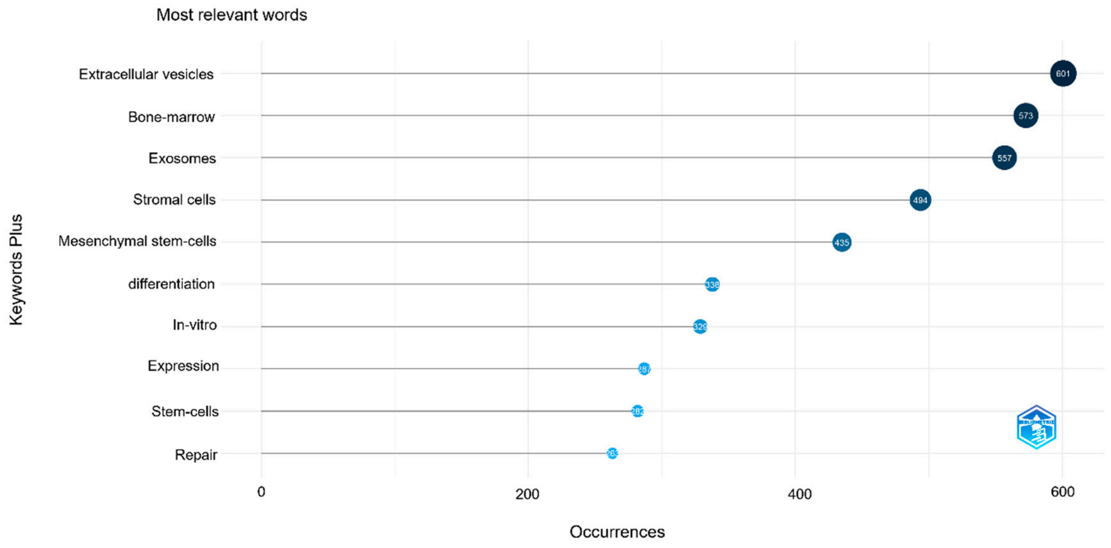

Figure S4 Most frequent words analyzed by Bibliometrix.

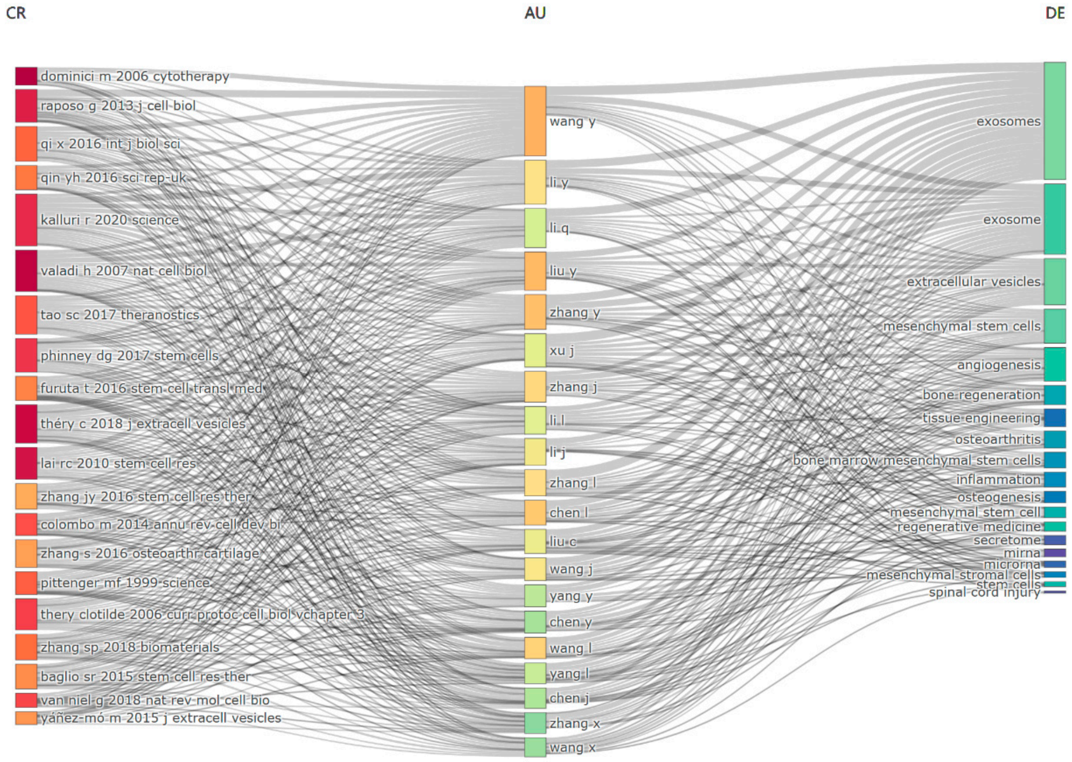

Figure S5 Three-field plot Sankey diagram by bibliometrix. Left: cited references; middle: Authors; right: Keywords

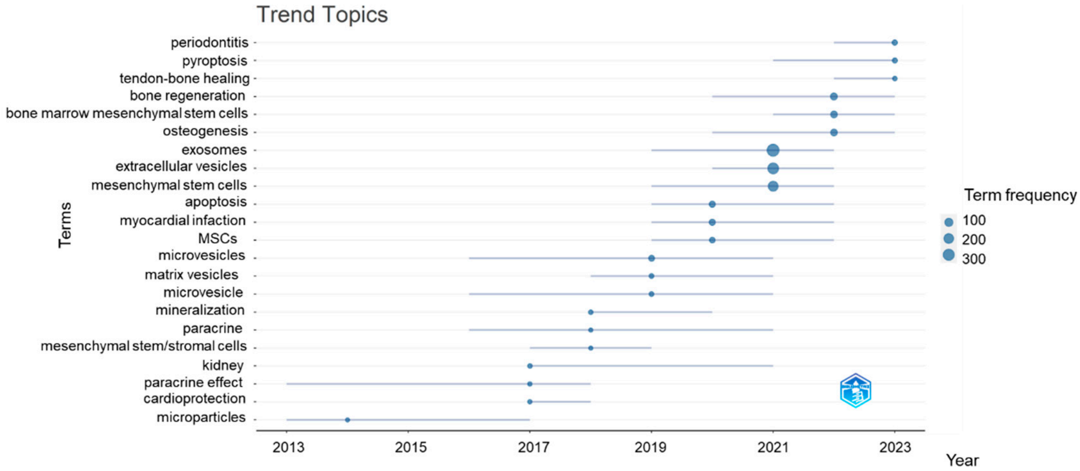

Figure S6 Trend topics analysis of keywords by Bibliometrix.
